# Development of a Biodegradable Subcutaneous Implant for Prolonged Drug Delivery Using 3D Printing

**DOI:** 10.3390/pharmaceutics12020105

**Published:** 2020-01-28

**Authors:** Sarah A. Stewart, Juan Domínguez-Robles, Victoria J. McIlorum, Elena Mancuso, Dimitrios A. Lamprou, Ryan F. Donnelly, Eneko Larrañeta

**Affiliations:** 1 School of Pharmacy, Queen’s University Belfast, 97 Lisburn Road, Belfast BT9 7BL, UK; sstewart35@qub.ac.uk (S.A.S.); vmcilorum01@qub.ac.uk (V.J.M.); d.lamprou@qub.ac.uk (D.A.L.); r.donnelly@qub.ac.uk (R.F.D.); 2 Nanotechnology and Integrated Bio-Engineering Centre (NIBEC), Ulster University, Jordanstown BT37 0QB, UK; e.mancuso@ulster.ac.uk

**Keywords:** implantable devices, subcutaneous, biodegradable, 3D printing, prolonged drug delivery

## Abstract

Implantable drug delivery devices offer many advantages over other routes of drug delivery. Most significantly, the delivery of lower doses of drug, thus, potentially reducing side-effects and improving patient compliance. Three dimensional (3D) printing is a flexible technique, which has been subject to increasing interest in the past few years, especially in the area of medical devices. The present work focussed on the use of 3D printing as a tool to manufacture implantable drug delivery devices to deliver a range of model compounds (methylene blue, ibuprofen sodium and ibuprofen acid) in two in vitro models. Five implant designs were produced, and the release rate varied, depending on the implant design and the drug properties. Additionally, a rate controlling membrane was produced, which further prolonged the release from the produced implants, signalling the potential use of these devices for chronic conditions.

## 1. Introduction

Implantable drug delivery devices are those that, when implanted into the body, release drugs at a defined rate and for a defined period. They offer advantages over other routes of drug delivery. They may achieve a therapeutic effect with lower drug concentrations [1,2,3] by potentially achieving higher drug concentrations at the site of interest, thus, reducing systemic drug exposure and minimising the potential for unwanted side-effects [4,5]. In addition, these devices allow personalised medicine, increased patient compliance [6] and prolonged delivery of treatment over weeks, months or years [7] in a device which may be removed if adverse effects require early termination of treatment [8,9]. Implantable delivery systems have been used for a range of clinical applications, most commonly contraception (e.g., Nexplanon^®^ and NuvaRing^®^) and cancer treatment (e.g., Vantas^®^) [3,10]. Nexplanon^®^ is a subcutaneous implant made from poly(ethylene vinyl acetate) which delivers etonogestrel over a period of three years before requiring removal [11,12]. Vantas^®^ is a subcutaneous implant made from a methacrylate-based hydrogel which delivers the drug histrelin for the treatment of prostate cancer over a period of one year [13]. Implantable drug delivery devices also have the potential to be used for other conditions such as the delivery of localised anaesthetics [14] or antibiotics [15].

Currently, the majority of implantable drug delivery devices which are available are manufactured from non-biodegradable polymers [10]. Thus, these implants require surgical removal once they have achieved their purpose. The surgical removal of non-biodegradable implants can often be more traumatic than their insertion [16]. Alternatively, biodegradable polymers offer the significant advantage of not requiring removal after their use, whilst still offering the potential for early removal, if required. They are designed to degrade naturally to products that can be excreted easily by the body [17]. Commonly used biodegradable and biocompatible polymers include poly(lactic acid) (PLA), poly(glycolic acid) (PGA), poly(lactic-co-glycolic acid) (PLGA) and poly(caprolactone) (PCL). Previously, these polymers have been successfully used in nanoparticle-based drug delivery systems and solid and microparticle parenteral implants [18] such as: Zoladex*^®^* (AstraZeneca, Cambridge, UK), a solid PLGA parenteral implant for the delivery of goserelin for the treatment of prostate cancer in men or breast cancer or endometriosis in women [19]; and Profact Depot*^®^* (Sanofi-Aventis, Paris, France), which is also a solid PLGA parenteral implant, for the delivery of buserelin. Other parenteral implantable systems use polymeric microparticles as the delivery carrier including: Sandostatin LAR*^®^* (Novartis, Basel, Switzerland) to deliver octreotide; or Risperdal Consta*^®^* (Janssen, Beerse, Belgium) to deliver risperidone [20]. 

The potential for personalisation of an implantable drug delivery device is substantial and becomes more likely due the increasing interest in 3D printing technologies. The high degree of flexibility and controllability of 3D printing would allow the preparation of tailored dosage forms with a release profile designed to exactly match the individual patient and condition to be treated [21]. Moreover, some of the disadvantages associated with 3D printing, such as high cost and speed, are improving as the technology becomes more widely used. The 3D printing approach to research newer (implantable) drug delivery devices can usher in a new era of treatments to various diseases.

The concept of drug delivery via an implantable device is not a new one. However, an implantable device that is cheap; easily manufactured; biodegradable; biocompatible and with a release rate that may be tailored to an individual patient, drug or clinical application is a very desirable goal, but one that is, as yet unachieved. 

Current research is often still focussed on the use of materials that are not biodegradable [22,23]. The aims of this study are 1) to develop 3Dprinted implantable devices for drug delivery using biocompatible/biodegradable materials and 2) to study the influence of the implant geometry on the drug release kinetics. For this purpose, we prepared different PLA and PVA implant designs using fused deposition modelling (FDM) 3D printing technology. These implants were designed containing “windows” of different sizes to allow drug release. Finally, a coating procedure using PCL was used to evaluate the possibility of obtaining a more sustained release from these implants. The resulting implants were characterised using different techniques such as X-ray micro-computed tomography and texture analysis. The last step was to evaluate the drug release kinetics from these implants by using different model molecules and two in vitro models.

## 2. Materials and Methods 

### 2.1. Materials

Granulate PLA (Ingeo™ Biopolymer 4043D) was purchased from NatureWorks (Minnesota, MN, USA). Filament PVA was purchased from Ultimaker (Ultimaker, Netherlands). Methylene blue, ibuprofen sodium, poly(ethylene glycol) (PEG) (M_W_ = 1000 Da), agarose powder and phosphate buffered saline (PBS) tablets pH 7.4 were purchased from Sigma-Aldrich (Dorset, UK). Sodium azide was purchased from Fluorochem Ltd. (Hadfield, UK). Ibuprofen acid was purchased from PharmInnova (Waregem, Belgium). Poly(caprolactone) (PCL) 6506 (M_W_ = 50,000 Da) and PCL 2054 (M_W_ = 550 Da) were provided by Perstorp (Perstorp, Sweden).

### 2.2. Methods

#### 2.2.1. Implant Designs

Hot-melt extrusion was used to produce the PLA filament, which would be used for the implant manufacture in combination with the PVA filament. PLA pellets were added to a filament extruder (3devo, Utrecht, The Netherlands) at an extrusion speed of 5 rpm and a filament fan speed of 70%. Finally, the temperature was adjusted through a control panel positioned at the side of the extruder, and it was between 170 and 190 °C, due to the existence of four heaters [24].

**Figure 1:**
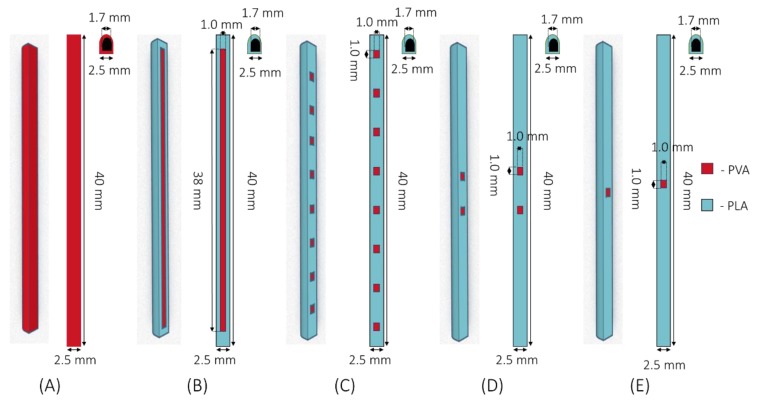
Schematic showing the implant designs: (**A**) 2.5 × 40.0 mm poly(vinyl alcohol) (PVA) implant; (**B**) 2.5 × 40.0 mm poly(lactic acid) (PLA) implant with one (1.0 × 38.0 mm) PVA “window”; (**C**) 2.5 × 40.0 mm PLA implant with eight (1.0 × 1.0 mm) PVA “windows”; (**D**) 2.5 × 40.0 mm PLA implant with two (1.0 × 1.0 mm) PVA “windows” and (**E**) 2.5 × 40.0 mm PLA implant with one (1.0 × 1.0 mm) PVA “window”.

Hollow implants were designed using a computer-aided design (CAD) software and printed using an Ultimaker3 3D printer (Ultimaker, Geldermalsen, The Netherlands) using Cura^®^ software. The Ultimaker3 system was equipped with two 0.4 mm extruder nozzles equipped with PLA and PVA, respectively. The print speed was 70 mm/s, the print temperature used was 205 °C, the build plate temperature was 85 °C and the layer height used was 0.2 mm. Five implant configurations were designed and produced (Figure 1A) 2.5 × 40.0 mm PVA implant (weight 0.15 ± 0.001 g); (Figure 1B) 2.5 × 40.0 mm PLA implant with one (1.0 × 38.0 mm) PVA “window” (weight 0.13 ± 0.007 g); (Figure 1C) 2.5 × 40.0 mm PLA implant with eight (1.0 × 1.0 mm) PVA “windows” (weight 0.13 ± 0.001 g); (Figure 1D) 2.5 × 40.0 mm PLA implant with two (1.0 × 1.0 mm) PVA “windows” (weight 0.14 ± 0.005 g) and (Figure 1E) 2.5 × 40.0 mm PLA implant with one (1.0 × 1.0 mm) PVA “window” (weight 0.14 ± 0.003 g). The thickness of the PVA “window” was 0.4 mm in all cases. Finally, implants were filled with a model compound by directly packing powder inside.

Finally, methylene blue (MB)-loaded implants (Figure 1B implant design) were coated with a formulation containing 50/50 PCL 6506/PCL 2054. This particular PCL composition was used because the coating of the implants with only PCL 6506 yielded implants that were not capable of releasing their MB cargo (data not shown). For this purpose, 5 g of this mixture was dissolved in 10 mL of dichloromethane (Merck, Darmstadt, Germany). Implants were coated following a dip-coating procedure using the previously prepared solution. The thickness of the resulting coating was measured using a digital calliper after pealing it from the implant. The coating showed a thickness of 0.11 ± 0.01 mm.

#### 2.2.2. Implant Characterisation

Optical coherence tomography (OCT) using an EX1301 OCT microscope (Michelson Diagnostics, Kent, UK) enabled visualisation of the dissolving PVA “windows” and the drug within the filled implant. The morphology of the implants was evaluated using electronic and optical microscopy. A Hitachi TM3030 benchtop scanning electron microscope (SEM) (Tokyo, Japan) and a Leica EZ4 D digital microscope (Leica, Wetzlar, Germany) were used. 

X-ray micro-computed tomography (μCT) scans were performed on 3D printed implants following the same methodology reported by Matthew et al. and Dominguez-Robles et al. [25,26]. Briefly, the 3D reconstruction volumes and inner structures of the implants were observed by using a Bruker SkyScan 1275 system (Bruker, Germany) with a Hamamatsu L11871 source. The microfocus of the X-ray source of the micro-CT scanner had a maximum voltage of 40 kV and maximum current of 250 μA. Samples were mounted vertically on dental wax and positioned 59.791 mm from the source, where the camera-to-source distance was 286 mm. No filter was applied for an exposure time of 49 ms. The images generated were 1944 × 1413 pixels with a resolution of 17 µm per pixel. Then the data were collected and Data Viewer as well as CT-An software were used to analyse them. Finally, CTVol software was applied to generate 3D reconstruction images.

The mechanical properties of the prepared implants were evaluated following a three-point bending test using a TA-XT2 Texture Analyser (Stable Micro Systems, Haslemere, UK). For all measurements the texture analyser was set in compression mode, with a cuboidal probe (9.5 cm in length) with a sharp end (1.1 mm thick) using a setup previously described by Donnelly et al. [27]. The probe was moved towards the implant at a speed of 0.5 mm/s. From the peak maximum of the force–distance curve, the break strength of each implant was calculated.

#### 2.2.3. Analytical Methods

Methylene blue (MB), ibuprofen sodium (IS) and ibuprofen acid (IA) were chosen as model compounds due to their different solubilities to assess any effect this may have on the release profiles. MB was quantified using UV spectroscopy (FLUOstar Omega Microplate Reader, BMG LABTECH, Ortenberg, Germany) at a wavelength of 668 nm. IS and IA were quantified using reverse-phase high-performance liquid chromatography (RP-HPLC) (Agilent 1220 series system, Agilent Technologies UK Ltd., Stockport, UK). The column used to achieve separation was Agilent Eclipse XDB-C18 (5 µm pore size, 4.6 × 150 mm) column (Agilent Technologies UK Ltd., Stockport, UK). The mobile phase used was composed of acetonitrile and 0.1% phosphoric acid at a ratio of 70:30, with a flow rate of 1 mL/min, injection volume of 50 µL and a sample runtime of 5 min. UV detection was carried out at 220 nm. The mobile phase was degassed by sonication for 30 min prior to use. The column temperature was regulated to 25 °C.

#### 2.2.4. In Vitro Drug Release Experiments

Implants were loaded with MB, IS or IA and placed in 500 mL of PBS (or PBS with 0.05% sodium azide for IS and IA release) at 37 °C and shaken at 40 rpm. Samples (0.5 mL) of the release medium were taken at specified time points and replaced with equal volume of PBS [28].

**Figure 2:**
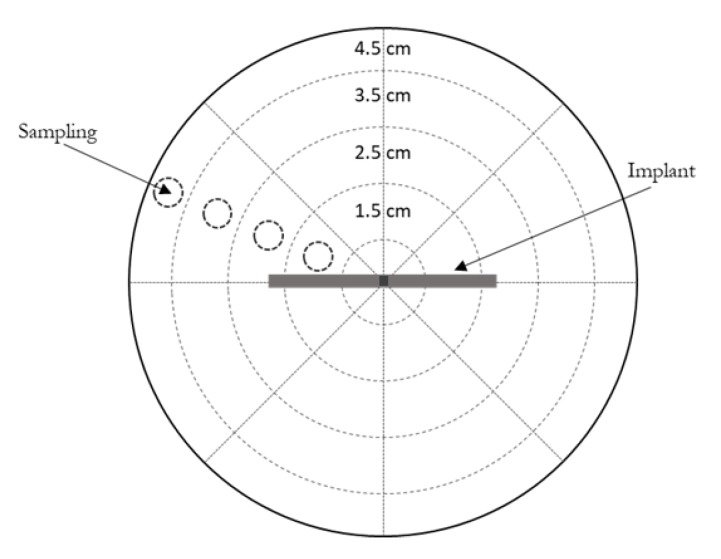
Schematic illustration of the in vitro experimental setup used to sample drug release into agarose gel.

As well as the agitated vessel in vitro release model, an agarose gel in vitro release model was also investigated to more closely mimic in vivo conditions [29]. Agarose powder was dissolved in PBS (for MB release) or PBS containing 0.05% of sodium azide (for IS release) and heated to prepare a 0.6% agarose solution. One-third of the required agarose solution was cast into a Petri dish (10 cm in diameter) and the implant (implant design E) was placed in the centre of this and the agarose solution was allowed to solidify. Subsequently, the remaining agarose solution was cast over this initial layer and allowed to solidify [29]. The Petri dishes were then covered with Parafilm M^®^, to prevent water evaporation, and placed into an airtight container within a non-agitated incubator at 37 °C. Cylindrical samples (0.5 cm diameter) of agarose were removed at predefined time points (Figure 2). Samples were weighed and analysed for their drug content using an appropriate method, as described in Section 2.2.3. Due to the symmetry of the agarose gel, it was assumed that the drug concentration was constant within each zone with the same distance from the implant “window” [29].

### 2.3. Data Analysis

Release profiles from each of the implants were compared by calculating and comparing the difference (*F*_1_) and similarity (*F*_2_) factor. *F*_1_ was calculated using Equation (1) that measures the percentage difference between two curves at each time point and is a measurement of the relative error between the two curves. Where, n is the number of time points, *R_t_* is the reference dissolution value at time *t*, and *T_t_* is the test dissolution value at time t [30,31].

(1) $$F_{1}=\left\{\;\left[\overset{n}{\underset{t=1}{\sum }}\left(R_{t}-T_{t}\right)\right]/\left[\overset{n}{\underset{t=1}{\sum }}R_{t}\right]\right\}\times 100$$

*F*_2_, shown in Equation (2), is a logarithmic transformation of the sum-squared error of differences between the test and reference products over all time points, *n*.

(2) $$F_{2}=50\times \log\left\{{\left[\left(1/n\right)\overset{n}{\underset{t=1}{\sum }}\left(R_{t}-T_{t}\right)\right]}^{-0.5}\times 100\right\}$$

In order for two dissolution profiles to be considered similar, the *F*_1_ value should be lower than 15 × (0–15) and *F*_2_ value should be more than 50 × (50–100) [30,31].

Where appropriate, all data were expressed as a mean ± standard deviation (SD) and compared using one-way analysis of variance (ANOVA) with Tukey’s post-hoc. In all cases, *p* < 0.05 was the minimum value considered acceptable for rejection of the null hypothesis.

## 3. Results and Discussion

### 3.1. Implant Design and Characterisation

**Figure 3:**
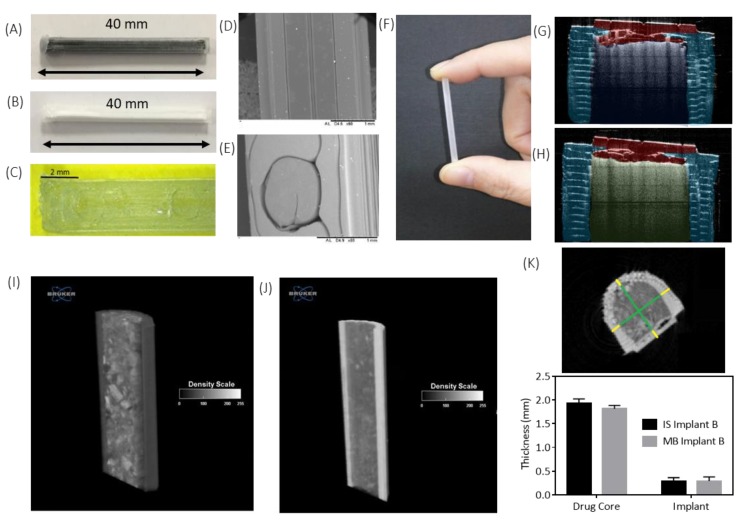
Images of (**A**) methylene blue-filled implant (Implant B); (**B**) ibuprofen sodium-filled implant (Implant B); (**C**) digital microscope image of a section of Implant C, (**D**) a scanning electron microscope (SEM) image of a section of a 38.0 × 1.0 mm poly(vinyl alcohol) (PVA) membrane, (**E**) SEM images of a 1.0 × 1.0 mm poly(vinyl alcohol) (PVA) membrane, (**F**) an image to show the size of the printed implant, (**G**) optical coherence tomography (OCT) image of an MB-filled implant and (**H**) OCT images of an IS-filled implant. Characterisation of implants through microCT analysis. Cross-section reconstructions in the *y*–*z* plane of the implants containing (**I**) MB and (**J**) IS. (**K**) Representative *x*–*y* cross-section of a 3D-printed implant used for quantitative analysis and dimensional measurements calculated at different locations over the implant 3D volume for the core/shell of the samples reported in (A) and (B), respectively.

A rod-shaped implant with a size of 2.5 × 40.0 mm was chosen because this shape and these dimensions are similar to dimensions that have already been shown to be acceptable in commercially available products and applicator devices have already been developed for an implant of these dimensions [32]. Implants were loaded with MB (68.6 ± 5.1 mg), IS (68.1 ± 3.0 mg) or IA (72.3 ± 3.2 mg). Images of the produced implants are shown in Figure 3A–H. These images give an appreciation of the actual geometry of the 3D printed “windows” in comparison to what was designed. Figure 3C, E shows that, although the 1.0 × 1.0 mm has been printed to the correct size, they are more circular in shape than square like the design. This is because the resolution of FDM printers is not as high as that displayed by other types of 3D printing such as stereolithography [33].

Implants A–E loaded with MB, Implants B and E loaded with IS and Implant B loaded with IA were tested using the agitated vessel release model. Implant E loaded with MB or IS was tested using the agarose gel release model. IA was not included in the agarose release model because of its poor solubility and the difficulties this would present to maintain sink conditions. These molecules were used due to their differing solubility values: MB 40 mg/mL [34]; IS 100 mg/mL [35] and IA 0.021 mg/mL [36]. These three molecules cover a wide range of hydrophobicity. Therefore, they are good candidates to establish how this parameter affects drug release from the 3D printed implantable devices. The influence of the solubility on the release profiles can be used to anticipate the release kinetics of other drugs loaded within the implants described here.

The architecture and topology of the 3D-printed implants were analysed using a Bruker SkyScan 1172 system μCT (Figure 3I–K). Cross-section reconstructions in the *y*–*z* plane of an implant containing (Figure 3I) MB and (Figure 3J) IS were performed, and representative *x*–*y* cross-section of a 3D-printed implant was used for quantitative analysis. These images (Figure 3I,J) give an appreciation of the drug distribution within the cavity of the implant and show that the drug distribution is uniform for both MB and IS. The dimensional measurements calculated at different locations over the implant 3D volume for the core and shell of the samples are reported in Figure 3K and show that there is no significant (*p* > 0.05) difference in the size of the drug core for either drug. This indicates that the drugs were dispersed through the entire implant cavity and that the packing process did not damaged the implant structure.

**Figure 4:**
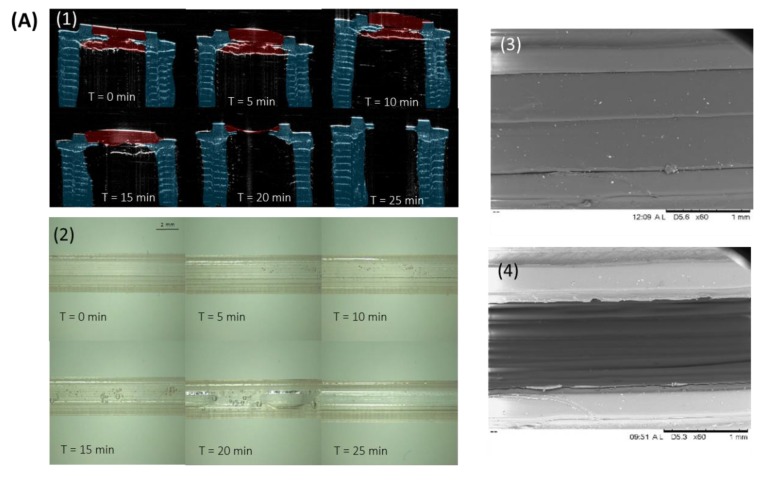

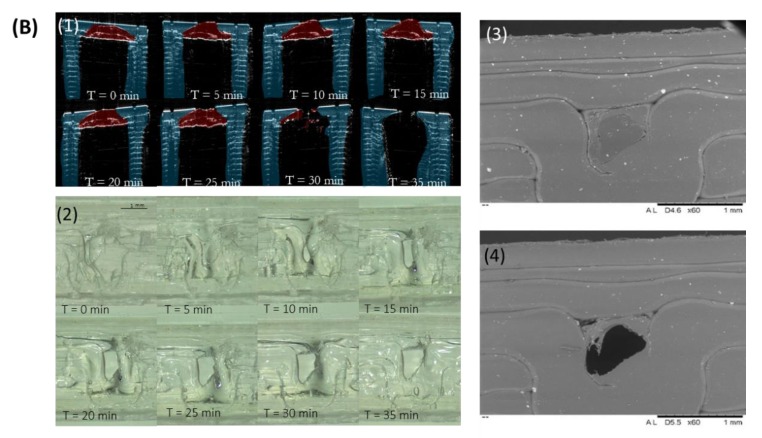
(**A**) Implant B (**1**) OCT images of poly(vinyl alcohol) (PVA) membrane dissolution in Implant B after emersion in phosphate buffered saline (PBS); (**2**) digital microscope images of poly(vinyl alcohol) (PVA) membrane dissolution in Implant B after emersion in PBS; SEM images of Implant B (**3**) before and (**4**) after dissolution. (**B**) (**1**) OCT images of poly(vinyl alcohol) (PVA) membrane dissolution in Implant E after emersion in PBS; (**2**) digital microscope images of poly(vinyl alcohol) (PVA) membrane dissolution in Implant E after emersion in PBS; SEM images of Implant E (**3**) before and (**4**) after dissolution.

Dissolution of the PVA “windows” in Implants B and E were visualised using OCT, digital microscopy and SEM and are shown in Figure 4A,B, respectively. It can be seen that complete dissolution of the PVA “window” in Implant B occurred after 25 min (Figure 4A1). Whereas, complete dissolution of the PVA “window” in Implant E took 35 min (Figure 4B1). Despite the “window” in Implant B being significantly larger than the “window” in Implant E, it fully dissolved more quickly. This may be explained by the reduced surface area-to-volume ratio of the “window” in Implant E, reducing the rate of dissolution for this implant. Goyanes et al. investigated the effect that the surface area-to-volume ratio had on the dissolution of PVA tablets and reported that a higher surface area to volume ratio resulted in tablets that dissolved more quickly [37]. It is important to note that the PVA “window” is designed to dissolve quickly to allow the drug to diffuse trough the generated “window”. The “window” material can be tailored to achieve a delayed drug release. Additionally, in the last section of the manuscript, an alternative coating approach was described to prepare implants allowing sustained drug release over months. It is important to note that a quick-dissolving commercial PVA filament was used for this study. PVA is a biocompatible polymer [38], but commercial filaments can have potential excipients, such as plasticisers, that are not ideal for medical applications. However, the present work is a proof-of-concept study exploring the influence of the structure of the implant on the drug release kinetics. Accordingly, a commercial PVA was used as it was the quickest approach. However, future work will require the use of filaments prepared using pure biocompatible polymer. This approach opens the possibility of developing implants with delayed release by printing the implant windows with polymers with slower dissolution/disintegration kinetics such as cellulose derivatives [39,40].

**Figure 5:**
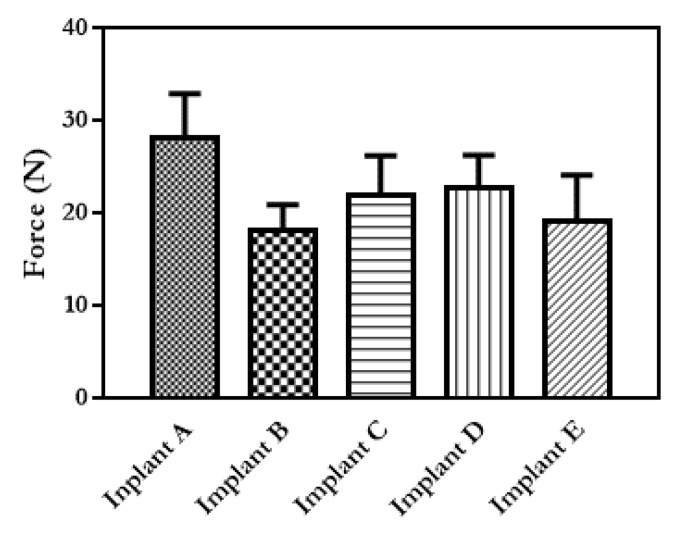
Force required to break each of the implant designs (*n* = 5, means + SD).

To predict robustness of the designed implants, their break strength and degree of flexibility were evaluated. A very rigid implant is likely to break during insertion or in situ; therefore, a degree of flexibility is required, as well as sufficient strength to withstand insertion and remain mechanically strong enough for the duration of drug release. If an implant breaks or cracks, it is likely to cause an increase or a burst in the rate of drug release which would, in turn, cause undesirable side effects in the patient. The maximum force required for breaking the implants and the angle of bending at the break point were calculated for each implant configuration and shown in Figure 5.

It can be seen from Figure 5 that there is no significant difference in the breaking force of Implants B–E (PLA implants). A significantly (*p* < 0.5) larger force was required to break Implant A (PVA), than was required for Implants B–E. This test was performed to evaluate if changing the design of the release “windows” from the implant has a direct influence on the mechanical properties of the resulting material. No mechanical tests directly comparable to those performed in this study have been performed on commercially available implantable drug delivery devices. However, mechanical testing of medical devices has been extensively reported. The results obtained here can be compared with the results reported by Horal et al. for 3D-printed PLA screws for orthopaedic applications [41]. In this case, PLA screws were manufactured and a three-point bending test was performed. The dimensions of these implants were similar to the ones described here (1–2 mm), and the forces applied during the bending tests were lower than the ones reported here (ranging between 0.5 and 10 N). These screws where designed for bone healing applications. Higher forces will be applied to bone screws than to implants designed to be implanted in soft tissue. Therefore, the implants presented here showed fracture forces higher than the forces that will be expected for soft tissue implants. As PLA has a long degradation time, up to 2 years [42], degradation of the implant structure would not be expected to have an effect on the mechanical properties during drug release or an effect on the release rate itself.

### 3.2. In Vitro Drug Release

**Figure 6:**
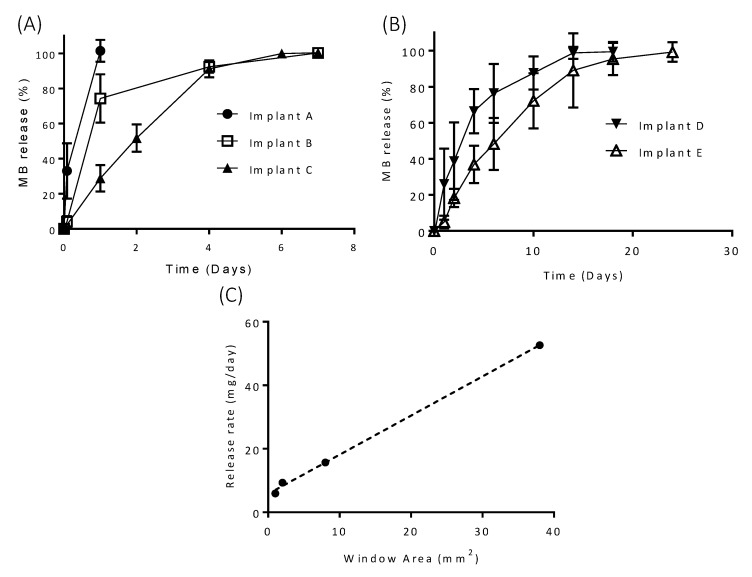
Release of methylene blue (MB) from (**A**) Implant A–C; (**B**) Implants D and E (*n* = 3, means ± SD) and (**C**) correlation between MB release rate and “window” area for the implants.

MB has some inherent antibacterial activity; therefore, bacterial growth in the release media was not anticipated to be an issue for these implants [43]. However, SA was added to IS and IA release media to prevent microbial growth [23,44,45] over the course of the release experiment. The release profiles of MB from each of the five implant designs are shown in Figure 6. Implants made entirely from PVA (Implant A) had the most rapid drug release, with 100% of drug releasing within 24 h. As expected, Implants B and C showed significantly extended release profiles in comparison with Implant A, with release time being extended to over six days. Although, Implants B and C took the same time to reach 100% release, Implant C showed a more sustained release profile, which showed less variation. Implants D and E showed an extended release profile in comparison to the other implants and show that reducing the size and number of “windows” effectively prolongs release from this type of implant. The release profiles of MB from each of the PVA “window” implants were compared using similarity and difference factor (*F*_1_/*F*_2_), and the results are shown in Table 1. Implant A had a significantly different release profile to Implants B and C as the F_1_ values were higher than 15 and the *F*_2_ values were lower than 50. Implants B and C and Implants D and E also showed significantly different release profiles to each other. These results indicate that implant design has potential to modify the release profile of a loaded molecule by simply changing the design of the implant. Interestingly, implants with 1.0 × 1.0 mm “windows” were capable of providing drug release over 25 days. A sustained release profile like this can be useful for local antimicrobial therapy or for pain management after surgery [46,47]. In these cases, a prolonged release over a period of a few weeks can be extremely beneficial to prevent infections or for pain management. However, for prolonged applications alternative approaches need to be evaluated. For this purpose, coated implants were evaluated. This approach is described in Section 3.3 of the present manuscript.

**Figure 7:**
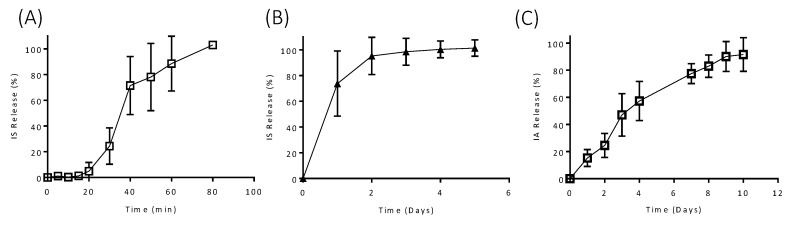
Release of (**A**) ibuprofen sodium (IS) from Implant B; (**B**) IS from Implant E and (**C**) release of ibuprofen acid (IA) from Implant B (*n* = 3, means ± SD).

The effect of drug properties on release from the designed implants was investigated by comparing the release profiles of MB (solubility 40 mg/mL [34]), with IS (solubility 100 mg/mL [35]) and IA (solubility 0.021 mg/mL [36]). The release profiles of IS from Implants B and E are shown in Figure 7A. The release rate of IS from Implant B was significantly increased in comparison to MB from the same implant. Complete IS release was achieved after just 80 min, whereas, 100% MB release took seven days. A similar increase in release rate is seen for Implant E, with 100% IS release achieved after six days and MB release after 25 days. These results show that obviously the implant design is not the only factor that contributes to change the release profile. The physicochemical properties of the drug loaded are important too. All in vitro releases were carried out under sink conditions; therefore, it is the dissolution rate of each of the drugs rather than solubility that is having an impact on drug release from the implant. Accordingly, changing the nature of the loaded molecule or including a formulation with a slower dissolution rate will provide an extra degree of control over the release profile. 

IA release from Implant B is shown in Figure 7C. The release of this compound is significantly extended in comparison with MB and IS release from the same implant design, with release taking ten days in comparison to six days and 80 min for MB and IS, respectively. As mentioned previously, the release rate of this drug is slower due to its slower dissolution kinetics, confirming that the nature of the drug loaded need to be carefully considered for each application type. 

**Figure 8:**
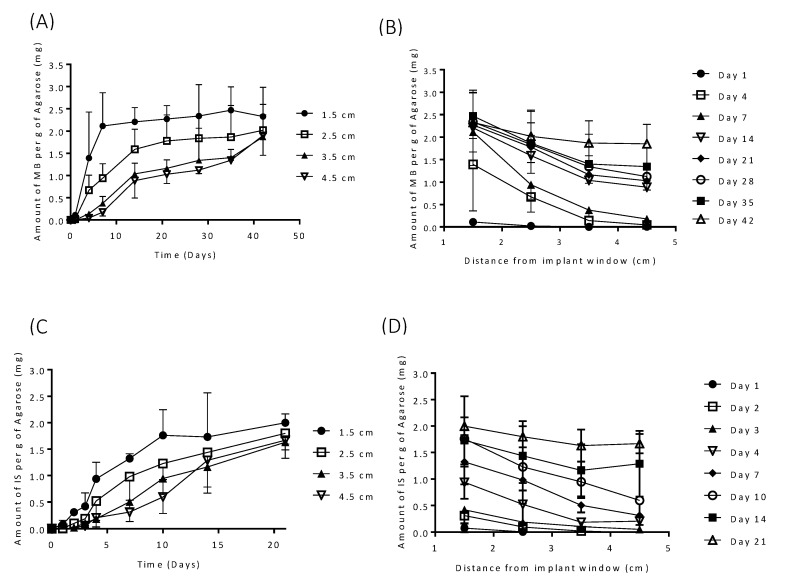
(**A** and **B**) MB and (**C** and **D**) IS releases from (**A**) Implant E into agarose gel (*n* = 3, means ± SD).

Figure 8 shows the release profiles of MB and IS from Implant E into an agarose gel release model. Release is expected to be slower in the agarose gel when compared to the agitated vessel release model. Within the agitated vessel model, convection rapidly homogenises the drug within the release media, thus, maintaining the drug concentration gradient at the interface of the implant with the release media. However, living tissues exhibit different conditions than those applied in the in vitro agitated vessel method. The extracellular matrix that these formulations are likely to be in contact with after implantation behave more like a gel than like a bulk fluid [48]. Despite the existence of a large number of biorelevant media for simulating physiological fluids, there is still not an accepted standard for simulation of subcutaneous environment [48]. Agarose gels form a 3D structure linked by hydrogen bonds with pore sizes similar to those encountered in physiological tissue and have been suggested as a more realistic in vitro release model than bulk fluid [29,49]. Moreover, multiple research works have reported the suitability of agarose hydrogel as a good release medium simulating soft tissues [50,51,52,53].

Both drugs demonstrated progressive drug release over a prolonged period. Figure 8A,B shows the release obtained for MB-loaded implants. These results showed that the closest region (1.5 cm) to the implant reached a plateau in MB levels after seven days. However, in further regions the MB concentration increased over time up to 40 days for the further regions (4.5 cm). This shows that MB was continuously delivered over 40 days. This MB concentration increase is not due only to MB diffusion through the agarose gel, as the concentration always increased. This suggests that there was a constant MB release that took place over time. After 40 days no significant differences were found in the release obtained at different distances from the implant (*p* > 0.05). This indicated that MB concentration all over the agarose gel was equivalent and that there was no concentration gradient that will drive more release. Similar behaviour was observed for IS (Figure 8C,D) over a period of 21 days. These results confirm that the testing conditions had a substantial influence on the release results. Moreover, this set of results suggest that the selected implants can be used to provide drug release over periods of several weeks. Similarly, Hoang et al. investigated releases of ciprofloxacin hydrochloride and vancomycin hydrochloride from bone implants over 48 and 96 h, respectively and showed that release into an agarose model was extended when compared to release of the same drugs from the same implants into an agitated vial [29].

The releases achieved in this work range from just 80 min to over 25 days in an agitated vessel and over 40 days in an agarose gel model and show promise as drug delivery systems for prolonged drug delivery. The use of local anaesthetics (commonly, bupivacaine, lidocaine and procaine) to treat localised pain has many advantages when compared with the systemic administration of opioids [14]. Work has been carried out to optimise the drug delivery of these agents to achieve localised delivery and limit peripheral side effects. An implantable device that could locally deliver anaesthetic over days or weeks could be of benefit for delivery of these drugs. Currently, the majority of chemotherapeutic agents are delivery systemically. This allows the drug to distribute throughout the entire body, including to healthy tissues, causing adverse side effects [54]. Polymeric devices aiming to locally deliver cancer drugs have been investigated and aim to improve the delivery of these drugs by providing localised sustained delivery and, therefore, reduce the effect on healthy tissue. Salmoria et al. investigated the use of polymeric implant to locally deliver fluorouracil and showed a desirable release rate over 45 days [54]. Localised delivery of antibiotics may offer advantages over conventional oral delivery for localised conditions. Gimeno et al. showed promising delivery of antibiotics which could be tailored by changing the implant design, from rapid drug release within 20 h to longer release times around 200 h for the potential prevention of orthopaedic-implant-associated infections [15]. These examples highlight instances where the implants developed in this work could be used.

### 3.3. In Vitro Drug Release from Coated Implants

The results described in previous sections show that these implants can be used for sustained drug delivery over periods of several weeks. The treatment of some medical conditions, especially chronic conditions, can be improved significantly with drug delivery devices capable of providing drug release over prolonged periods of time. These periods of time range from months up to years for potent compounds such as hormones. Examples of this will be the treatment of chronic conditions or even pre-exposure prophylaxis of human immunodeficiency disease (HIV). 

**Figure 9:**
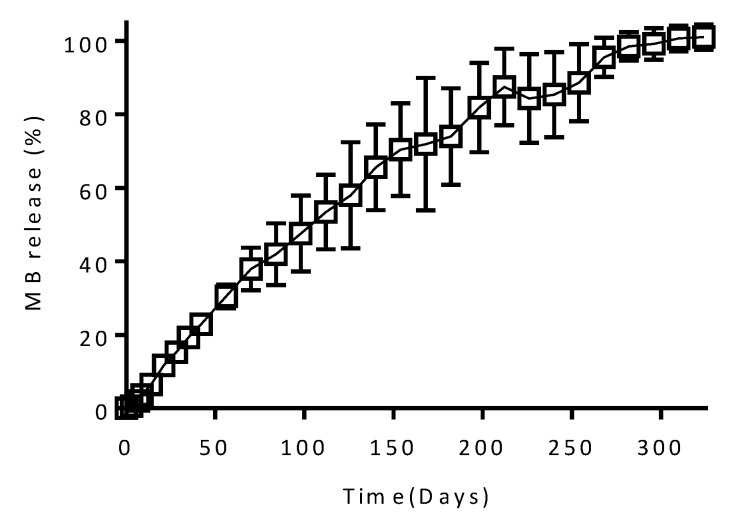
Release profile of methylene blue from Implant B with a PCL formulation coating (*n* = 3, means ± SD).

A good alternative to obtain implants with prolonged drug release profiles is to coat them with a membrane capable of sustaining drug release [55]. Accordingly, a simple dip coating procedure can be used to prepare implants with prolonged drug release profiles. Accordingly, a thin film covers the surface of the implant acting as a rate controlling membrane [9]. Figure 9 shows the release profile of MB from implants (Implant B) coated with a PCL-based formulation. It can be seen that the PCL rate controlling membrane is capable of providing sustained drug releases over periods of 300 days. Interestingly, non-coated equivalent implants showed MB release profiles extended over only four days (Figure 6). These results suggest that PCL coating could be an ideal approach for applications that require drug release over longer periods of time. PCL has been described previously as a good candidate to prepare rate controlling membranes for drug delivery applications [9]. PEG membranes have been used before to release tenofovir alafenamide for HIV pre-exposure prophylaxis [56]. These systems achieved prolonged releases between 100 and 200 days. Considering that tenofovir alafenamide shows a lower water solubility than MB, the system described has great potential for sustaining the release of hydrophilic molecules as MB showed up to 300 days of release.

## 4. Conclusions

In this work, hollow 3D-printed implants with similar dimensions to those already available in the market were successfully produced. The flexibility of this manufacturing technique allowed five different implant designs to be easily designed and produced. This technique has the potential to allow personalisation of implantable drug delivery devices for individual patients and conditions. μCT confirmed consistent drug distribution within the implant and confirms the implants’ suitability for a range of drug compounds. The mechanical properties of the designed implants were superior to those of other drug delivery systems. This work has shown that the release rate from these implants can be modified by changing the implant design but is also dependent on the properties of the compound contained within the implant. Finally, implant coating can provide an added degree of control over the release, with a PCL-based coating showing potential to extend expressively the release profile.

The results described in the present work demonstrate how 3D printing is a promising technology for drug eluting implant manufacture. Considering the simplicity of the technology described here, it can be easily transferred to a clinical setup, where implants could be designed on demand to fulfil patient’s needs after surgery. These implants may be suited for delivery of drugs for localised treatment. For example, chemotherapy agents, antibiotics or localised anaesthetics. Alternatively, they could be tailored by coating them for prolonged drug delivery for the treatment of chronic conditions. This can be done due to the versatility of 3D printing technology.

## Figures and Tables

**Table 1:** Difference (*F*_1_) and similarity (*F*_2_) factor of each release profile for methylene blue (MB) release from poly(lactic acid) (PLA) implant with poly(vinyl alcohol) (PVA) “window” designs.

Curve 1	Curve 2	*F*_1_	*F*_2_
Implant A	Implant B	60.06	33.00
Implant A	Implant C	73.89	13.58
Implant B	Implant C	28.93	32.12
Implant D	Implant E	19.61	34.75
